# Evaluating the Co-design of an Age-Friendly, Rural, Multidisciplinary Primary Care Model: A Study Protocol

**DOI:** 10.3390/mps5020023

**Published:** 2022-03-07

**Authors:** Rachel Winterton, Kathleen Brasher, Mark Ashcroft

**Affiliations:** 1 John Richards Centre for Rural Ageing Research, La Trobe Rural Health School, La Trobe University, Bendigo, VIC 3550, Australia; 2 Upper Hume Primary Care Partnership, Wodonga, VIC 3690, Australia; kathleen.brasher@upperhumepcp.com.au; 3 Beechworth Health Service, Beechworth, VIC 3747, Australia; mark.ashcroft@beechworthhealth.org.au

**Keywords:** age-friendly, older people, primary care, co-design

## Abstract

In the context of increased rates of frailty and chronic disease among older people, there is a need to develop age-friendly, integrated primary care models that place the older person at the centre of their care. However, there is little evidence about how age-friendly integrated care frameworks that are sensitive to the challenges of rural regions can be developed. This protocol paper outlines a study that will examine how the use of an age-friendly care framework (the Indigo 4Ms Framework) within a co-design process can facilitate the development of models of integrated care for rural older people within the Upper Hume region (Victoria, Australia). A co-design team will be assembled, which will include older people and individuals from local health, aged care, and community organisations. Process and outcome evaluation of the co-design activities will be undertaken to determine (1) the processes, activities and outputs that facilitate or hinder the co-design of a 4Ms integrated approach, and (2) how the use of the Indigo 4Ms Framework within a co-design process contributes to more integrated working practices. This protocol contributes to the development of a field of study examining how rural health and aged care services can become more age-friendly, with an emphasis on the role of co-design in developing integrated approaches to health care for older adults.

## 1. Introduction

Global population ageing trends are presenting significant challenges associated with maintaining the health, wellbeing and functional capacity of older people [1]. For example, international literature highlights that chronic disease is a significant challenge to the health of older people, with chronic conditions related to sixty per cent of disability-adjusted life years [2,3]. In 2017–2018, four in five Australians aged sixty-five years and over had one or more chronic conditions [4]. Chronic illness is associated with greater complexity of health needs, multiple hospitalisations, a poorer quality of life, and the early onset of functional decline [5,6]. At the same time, the risk of frailty among community-dwelling older people is increasing [7], which reflects a state of increased vulnerability to adverse health outcomes. Frailty is associated with declines in reserve and function related to biological, psychological, and social factors [7]. It is a risk factor for falls, dependency, hospitalisation, and mortality, and is associated with higher health and aged care costs [8].

However, Western health systems prioritise episodic, short-term, fragmented and curative approaches to care [8,9], with geriatric care often focusing on specific diseases. This overlooks common difficulties in hearing, seeing, moving and remembering that arise from the gradual biological changes that accompany ageing, leading to a gradual decrease in physiological reserve [6]. To prevent this decline and maintain the health, wellbeing and functional ability of older people, international health policy is advocating for integrated, patient-centred primary care responses as a fundamental component of health system reform [6,8,10]. Integrated care seeks to overcome the fragmentation of care by linking or co-ordinating the services of providers along the continuum of care [11], with the patient-centred component reflecting a need to tailor this integration to individual needs and preferences [12]. Integrated care aims to improve health outcomes while delivering a higher quality service to patients, lowering costs and ensuring the wellbeing of the health workforce [8].

The implementation of integrated care for older people has generally been poor, with limited evidence of its effectiveness [13]. Specifically, there is an absence of evidence-based policy and systems reform targeting older people [14,15], impeded by the complexity of integrated care itself within a complicated health system [8]. Funding silos, competition between services—public, private, health and social—a lack of long-term policy commitment and strong leadership have all been identified as factors for the stalling of reform [8,16]. The World Health Organization (WHO) calls for the restructuring of health services to improve care for older people by placing them at the centre of service delivery. Practically, this means that health care is organised around their needs and preferences, and design for integration across service levels and types [6]. The WHO Integrated Care for Older People (ICOPE) guidelines [10] provide evidence-based direction and interventions to enable an older person to maintain, slow or reverse any declines in their physical and mental capacities. Concomitantly, the Institute for Healthcare Improvement (IHI), in partnership with the John A. Hartford Foundation, the American Hospital Association, the Catholic Health Association of the United States and leading geriatric care experts, co-designed a measurable, feasible and sustainable approach to make the U.S. health care systems age-friendly [17]. The IHI Age-Friendly Health System Framework comprises four evidence-based core elements known as the ‘4Ms’ with associated high-level, evidence-based interventions (What Matters, Mobility, Medications and Mentation). There is evidence that the application of the 4Ms Framework improves physical and psychosocial outcomes for older people within health settings while reducing harm and costs [18].

However, there is little evidence relating to the applicability and implementation of such integrated age-friendly care models in rural contexts [19]. Globally, the proportions of older people residing in rural locations are increasing, demonstrating a need for care models to be age-friendly. However, when compared with urban settings, rural residents have higher mortality and co-morbidity rates, higher rates of suicide and injury, a higher burden of disease and experience more potentially preventable hospitalisations [20,21,22,23]. In general, rural residents also experience poorer levels of access to and use of health and aged care services when compared with their urban counterparts [21,22,23]. Global research has highlighted significant resource challenges associated with delivering care to older people in rural settings when compared with urban regions. Rural regions have lower levels of access to health, specialist and support services, and have experienced greater levels of centralisation, withdrawal and standardisation of health and aged care services, which has led to more regionalised service delivery approaches that can reduce health and aged care service availability in rural regions [23,24,25,26,27]. Additionally, rural health providers experience financial pressures associated with service delivery to geographically isolated rural regions [25], and challenges in recruiting and retaining an adequate and qualified rural health and aged care workforce [28]. Consequently, understanding how to design appropriate age-friendly health care models in rural settings specifically is important in the context of these increased levels of demographic need, different patterns of engagement with health and aged care services, and potential differences in health and aged care workforce availability, capacity and access.

To address this, some emergent work is being undertaken in the United States [29] and Australia [19,30], with specific rural jurisdictions seeking to adapt the existing 4Ms framework to reflect local conditions. In Australia, a rural-specific model that aligns with national and local rural health priorities and conditions, existing accreditation standards and evidence-based care practices, has been developed using the IHI 4Ms Framework as a guide [30]. The eventual framework, the Indigo 4Ms Framework (The Framework), was developed by local rural health experts, consumers and other end-users using the Australian National Health and Medical Research Council (NHMRC)’s advice on the development of guidelines. The Framework encompasses the IHI Framework and WHO guidelines through four domains and their related aims, which encompass 12 core elements and 34 associated key actions:

What Matters: provide person-centred assessment and care planning.
Medication: eliminate unnecessary, ineffective and duplicative medicines.
Mobility: improve musculo–skeletal function and mobility.
Mental Wellbeing: promote psychological wellbeing and prevent cognitive impairment.

The Indigo 4Ms Framework provides a cohesive structure for the care of older people, regardless of the care setting and level of functional ability of the person. This enables a single framework to be used for health assessments and prevention across the rural care continuum, from hospital-level clinical interactions through to rural community care services. The Framework operates as a heuristic device, translating large amounts of information into a quick mental reference to structure and prioritise clinical interactions [30]. This enables both rural providers and patients to determine what matters most when deciding on actions, paradoxically leading to a ‘less–is–more effect’, where less information leads to greater accuracy [31].

However, there is little international evidence relating to how a 4Ms approach to age-friendly care can actually be used to guide the design of rural models of age-friendly integrated care at a regional level. This is particularly important in the context of continued centralised, regionalised approaches to rural primary and geriatric care delivery [26,27]. Specifically, there is a need to identify the processes, activities and outputs that support or inhibit the co-design of an integrated model for age-friendly care in rural contexts, in order to understand some of the potential enablers and barriers to the co-design of age-friendly care models in rural settings. This paper outlines a protocol for evaluating how the use of the rural-specific Indigo 4Ms framework within a co-design process can facilitate the development of regional models of integrated care within rural settings.

### Aim and Objectives

This research aims to determine how the use of the Indigo 4Ms Framework can facilitate the development of models of integrated care for rural older people within the Upper Hume region through a co-design process. To do so, it will address two key questions:

1. What are the processes, activities and outputs that facilitate or hinder the successful co-design of a 4Ms integrated approach to the care of older people?
2. How does the use of the Indigo 4Ms Framework within a co-design process contribute to more integrated working practices among multi-disciplinary rural health and aged care workforce and community organisations?

## 2. Methods

### 2.1. Study Design

This study will employ both process and outcome evaluation methods [32] to evaluate how the use of the Indigo 4Ms Framework within an experience-based co-design (EBCD) process can facilitate models of integrated care for rural older people within the Upper Hume region. EBCD is a participatory research approach that draws upon design tools and ways of thinking in order to bring healthcare staff and consumers together to improve the quality of care [33].

### 2.2. Setting and Participants

This project will be undertaken within the Upper Hume region of northeast Victoria, Australia. This region is located on the border of two Australian states (Victoria and New South Wales), approximately three hours north of Victoria’s capital city, Melbourne. It encompasses the regional centre of Albury/Wodonga, and the local government areas (LGAs) of Indigo, Towong and Wodonga. This region is home to one large regional hospital (Albury Wodonga Health), three small rural health services and a series of community health services. Under the Modified Monash Model (MMM) categorisation, which is used to classify regions from a health workforce perspective and reflects the level of remoteness and population size, the region is primarily classified as MMM5–small rural [34].

The participants of the study will be involved in the administration or provision of primary care services to older people in the Upper Hume region or be engaged in the direct receipt of primary care services to older people. Primary care is provided in community settings and refers to medical care provided by health care professionals, such as (but not limited to) general practitioners, nurses, dentists, pharmacists, allied health and mental health providers, and Aboriginal and Torres Strait Islander health practitioners [35].

### 2.3. Inclusion Criteria

For inclusion in this study, participants will be aged 18 years and above, and be:

- Members of the Project Control Group (PCG) steering the project, who are comprised of representatives from local hospitals, rural health services and community health services and primary care partnerships;
- Health care professionals involved in the administration or provision of primary care services to older people within the Upper Hume region;
- Community-dwelling older people (aged 65+), or carers of older people who reside in the Upper Hume region.

### 2.4. Intervention

The co-design activities will follow the four stages of co-design developed by the New South Wales Agency for Clinical Innovation [36] for successful co-design in health:

- Stage 1 (Engage): Frame the problem/opportunity and build a team of people who use or deliver health care;
- Stage 2 (Understand): Develop co-design preparedness by developing knowledge of co-design processes and learning from/gaining an understanding of lived experiences;
- Stage 3 (Gather): Identify touchpoints and opportunities for improvement, prioritise together;
- Stage 4 (Improve): Design improvements, test, learn and repeat.

At Stage 1 (Engage), a co-design team will be assembled. This co-design group will involve practitioners and individuals from local health, education, aged care, and community organisations across the Upper Hume region. These may encompass, but will not be limited to, people from the following organisations or groups:

- Health: acute, sub-acute and community health professionals from local health and community services within the Upper Hume region, local GPs and pharmacists;
- Education: local institutions involved in the education of health and aged care professionals;
- Aged Care: Representatives from organisations engaged in the provision of home-based aged care services;
- Community: representatives from health services’ Community Advisory Committees, local older people’s organisations (e.g., Senior Citizens, U3A, carer support groups) and older people living in the communities in the Upper Hume region.

Approximately 30 representatives will be sought, consistent with other health co-design projects [37]. Where possible, diversity within the sample will be sought in relation to organisational representation, community spread and disciplinary background.

Individuals will be recruited using the following mechanisms:

- A study information form will be circulated by the local Primary Care Partnership by email to their mailing list of local health, education, government and community stakeholders. A Participant Information and Consent Form (PICF) explaining the co-design process and the information that will be collected within the co-design process and evaluation will also be circulated with this study information form;
- Collaborating health organisations will be asked to circulate the study information form to their Consumer Advisory Committees, with the PICF;
- Community members will be encouraged to ‘bring a friend’ by means of providing the contact details of the research team to interested community members. If contacted, the research team will provide a copy of the study information form and PICF, explaining the co-design process and the information that will be collected within the co-design process and evaluation;
- Key health organisations within the Upper Hume (as listed in the collaborating organisations within the PICF) will also be asked to forward information on the project (the study information form and PICF) within their organisations to people that they feel may be able to contribute;
- A list of publicly available contact details for health, aged care and community organisations not captured using the above mechanisms will be compiled by the research team using Google and local community directories. An expression of interest form and PICF will be circulated to these contacts introducing the project.

Participants will be asked to contact the project manager by email to express their interest, at which point the project manager will contact them to:

- Ensure that they understand what they are consenting to;
- Provide any additional detail required;
- Arrange completion of the consent form;
- Ask them to fill in a short expression of interest form once the consent form has been returned, which will ask them for their name, type of representative (practitioner/provider/community representative), current organisation and position and local government areas they work across, or live in (older people/carers only).

This expression of interest form can be returned either to the project manager by email, or in-person at the first workshop. Within the PICF and promotional information, it will be emphasised that participants will not be acting as representatives of their organisation, but will be drawing on their individual/practice expertise in the care of older people.

### 2.5. Study Procedures

#### 2.5.1. Co-design Group Members

Co-design participants will be required to participate in and contribute to a series of co-design workshops (held over a 1.5-year period), which will each be approximately 3 h in duration. These workshops will be held at a local health service, and will encompass:

- Workshops 1 and 2: (Stage 2—Gather): Training workshops on (1) co-design and the Indigo 4Ms Framework; and (2) interactive co-design workshops exploring the lived experience of older people and carers in relation to health care, and the delivery of current health and social care consistent with the Indigo 4Ms across the region. Within the second workshop, specific attention will be directed towards how rural settings impact the lived experience of older people and current delivery models. Written notes from the second co-design workshop will be typed into Microsoft Word and entered into a qualitative data analysis program (NVivo) and analysed thematically using inductive methods against the following key questions for the workshop: (1) experiences of older people and carers accessing care within the Upper Hume region, and (2) current examples of health and social care consistent with the Indigo 4Ms being delivered within the Upper Hume region (what matters, medication, mental wellbeing and mobility). The role of rurality in structuring or influencing these experiences and examples will also be interrogated across both of these key questions. Braun and Clarke’s [38] framework for qualitative thematic analysis will be used to guide the analytic process using NVivo within the co-design phases. Here, two independent data coders will review the data and generate the initial codes in relation to the key questions. At this point, themes within these codes will be generated and reviewed in relation to other themes and the entire dataset to ensure consistency and rigour. Part of this process will include cross-checking of themes between coders to ensure consensus. This will generate a report on the lived experiences of older people and carers within the Upper Hume region, with specific reference to rural enablers and challenges, which will then be critically interrogated by the co-design group at Workshop 3 to identify potential gaps and priorities for the Indigo 4Ms model of care;
- Workshop 3 (Stage 3—Understand): Synthesis of data gathered at Stage 2 to identify gaps and duplication of services, current best practices relating to culturally and locally appropriate care, opportunities for collaboration and priorities for the models of care, appropriateness of existing tools and resources. Within this workshop, the role of rural contexts in shaping these factors will also be considered. Written notes from this co-design workshop will be used by the research team to develop a prototype of an Indigo 4Ms model of care, which will have been outlined by the co-design team within their notes, and this will be interrogated by the co-design team at Stage 4;
- Workshops 4 and 5 (Stage 4—Improve): Testing and co-designing the Indigo 4Ms models of care and implementation plan developed at Stage 3, and tools and resources to guide quality improvement processes. Written notes from Workshop 4 will be typed into Microsoft Word and entered into a qualitative data analysis program (NVivo), and analysed using thematic deductive analysis [38] in accordance with the strengths, weaknesses, opportunities and threats relating to the proposed Indigo 4Ms model of care within their specific rural community settings. These findings will then be provided back to the co-design team, who will use these to further refine the prototype model of care and develop recommendations/training for implementation.

Prior to the commencement of the first co-design group, co-design group participants will be invited to sign a consent form agreeing to participate. Consent will be re-confirmed by a member of the research team prior to the commencement of each workshop, with a summary of the nature of the information to be collected at each workshop provided to guide informed consent. Participants will be free to withdraw from active participation at any point, and this will be affirmed at the commencement of each workshop. However, given the grouped, anonymised nature of the data, they will be unable to withdraw data that has already been provided. Older people and carers who participate in the project will receive a $40 voucher for each workshop they attend to offset costs associated with participation.

Within the co-design group activities, the following data will be collected by the research team to assist in process and outcome evaluation:

- Anonymised, aggregated demographic data provided within the expression of interest form to provide a general participant profile of the co-design group (e.g., community they work in, clinical background and sector of work/community representation);
- Anonymised, grouped attendance data from each workshop (numbers of attendees and community, clinical background and sector of work/community representation);
- Minutes from co-design workshops and written notes generated by the group within the co-design workshops relating to project data, with no data identifying individuals in any way (any identifying data related to individuals will be removed by the project manager prior to giving it to the research team);
- Field notes collected by the research team during their attendance at these activities;
- Outputs developed through co-design activities (e.g., reports).

#### 2.5.2. Project Control Group (PCG) Participants

The co-design process will be overseen and governed by the PCG, comprised of the CEOs or delegates of the members of the local health services involved in the project, as well as two community representatives. This group will meet once per month during the project. Community representatives involved in the PCG will have their out–of–pocket and travel expenses covered. The following sources of data will be collected to inform process and/or outcome evaluation:

- Ratified project management documents (project plans, project governance plans and terms of reference);
- Meeting agendas and minutes from project control group (PCG) meetings;
- Written reports and outputs generated throughout the project that have been ratified by the PCG.

Members of the PCG will be subject to a separate process of gaining informed consent that will support the use of these sources of data. At the formal commencement of the project, members of the PCG will be provided with a Participant Information and Consent Form (PICF) that specifically states that meeting agendas and minutes will be provided to the research team for use in process evaluation. PCG members will also be verbally advised at the commencement of each meeting that the agenda and ratified minutes will be provided to the research team to assist in process evaluation as a matter of standing business. Participants will be asked to sign a consent form acknowledging that their contributions that are recorded in meeting minutes and agendas will be provided to the research team for use in analysis. The consent form will be required to be returned to the project manager, either in person or by email. Any participants who do not feel comfortable in consenting to this will be encouraged to contact the project manager to ensure that they can still contribute to the project. This may encompass not recording the data they provide specifically within project minutes and including them as an addendum that will not be subject to analysis. However, it will be reiterated that meeting minutes will not attribute information to individuals unless with the express permission of that individual (which will be gained through the ratification of meeting minutes). All this information is provided to participants within the PICF.

### 2.6. Outcomes

This intervention and the process and outcome evaluation that will accompany the intervention will yield two primary outcomes:

1. Development of recommendations to support other rural regions to co-design age-friendly rural health services by means of identifying the processes, activities and outputs that support or hinder successful co-design activities using the Indigo 4Ms approach in rural contexts. This will assist in identifying enablers and barriers to designing, planning and delivering rural age-friendly care. This will feed directly into other work being completed to design 4M approaches to health care, both in Australia and internationally [18,39];
2. Identification of how, or whether, the use of the Indigo 4Ms within a rural primary care co-design process can facilitate more integrated working practices or attitudes among rural health, education, aged care and community stakeholders. This will provide evidence relating to how the use of the model may act as a change agent in restructuring practices and attitudes relating to rural patient-centred primary care integration. It may also provide evidence relating to its limitations within a rural primary care setting.

### 2.7. Data Analysis

**Figure 1:**
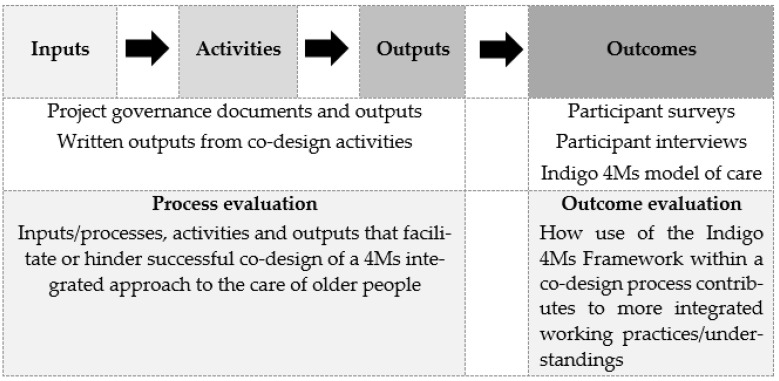
Program logic.

Analyses for the process and outcome evaluation of the intervention will follow the program logic outlined in Figure 1.

#### 2.7.1. Process Evaluation

To determine the processes, activities, and outputs that facilitate or hinder the successful co-design of a 4Ms integrated approach to the care of older people, project governance documents and co-design activity documents and data will be collated and analysed to explore the three key components of process evaluation within the project’s logic model: inputs, activities and outputs (see Table 1).

All data will be entered into a qualitative data analysis program (QSR-NVIVO) or into an Excel spreadsheet (categorical/numerical data) with data analysed thematically using the Braun and Clarke [38] framework in relation to the following questions:

- Inputs: What was contributed and by whom;
- Activities: What activities were completed and when/where/why/how;
- Outputs: What was generated.

These initial thematic findings will then be critically analysed using the following higher-order questions:

- Inputs: How inputs changed or evolved over the project;
- Activities: How activities aligned with project plans, and how the Indigo 4Ms were discussed within project activities;
- Outputs: How outputs aligned with project plans, and how outputs reflected the Indigo 4Ms approach.

These inputs, activities and outputs will also be critically assessed using deductive thematic analysis against Greenhalgh et al.’s [40] elements of successful co-creation in health, which encompass the presence of a systems perspective, the framing of research as a creative enterprise with human experience at its core, and emphasis on process.

#### 2.7.2. Outcome Evaluation

To determine how the use of the Indigo 4Ms Framework within a co-design process contributes to more integrated working practices among multi-disciplinary rural health and aged care workforce and community organisations, the following methods will be employed:

##### Survey of Co-design and PCG Group Members

An online survey (using the QuestionPro survey platform) will be administered to all members of the co-design group and PCG via email by the project manager to determine how their perspectives on current approaches to integrated care for older people within the region change over the course of the intervention. The survey will be administered at two points of the project (prior to Workshop 1—Survey 1, and at the conclusion of Workshop 5—Survey 2), with participants given 2 weeks to complete the survey. These surveys will not be linked in any way. Participants will be asked to respond to the following sets of questions, which will take approximately 30 min:

Contextual data:

- Participant demographic information (gender, sector of work/involvement and local government area in which they live/work);
- Number of workshops attended (Survey 2 only);
- Status as participant—current or past (Survey 2 only);
- If past participant, reasons they are no longer participating.

Outcome data:

- Five-item Likert scale questions derived from the validated Project Integrate framework [41], which provides seven dimensions and forty items associated with perspectives on the successful implementation of integrated care, which encompass person-centred care (7 items), clinical integration (7 items), professional integration (5 items), organisational integration (5 items), systems integration (6 items), functional integration (4 items) and normative integration (6 items).

A PICF will be attached to the recruitment email detailing the information sought and what the information will be used for. Consent will be implied through the return of the survey, and participants will be asked to provide an acknowledgement that they have read the PICF and are aware of how their information will be used. All responses will be anonymous and no identifying information will be sought. Participants will be notified in the PICF and at the commencement of the survey that, as the responses are anonymous, data will not be able to be withdrawn once the survey has been completed. However, they will be able to cease completing the survey at any time by closing their browser.

At the conclusion of both survey 1 and survey 2, anonymised survey data will be exported from QuestionPro into a quantitative data analysis program (SPSS) and data cleaning will take place. At both survey 1 and 2, the following analyses will be completed:

- Descriptive statistics will be generated for participant characteristics: gender, age, community representation, type of participant (older person/carer/community member/practitioner), number of workshops attended (Survey 2 only) and status as a participant (Survey 2 only);
- Mean, minimum and maximum scores and standard deviation will be calculated for each of the 40 items to determine the current perspectives on integrated care for older people within the region;
- At Survey 2, the mean, minimum and maximum scores and standard deviations for each of the 40 items will be compared against the findings from Survey 1 to determine if and how/in what areas perspectives on integrated care for older people within the region have changed. This comparison will be descriptive in nature, rather than using inferential statistics.

##### Follow-Up Interviews with Selected Co-design Group Members

At the conclusion of Survey 2, survey participants will be asked if they are willing to participate in a short follow-up interview by phone with a research team member. If they are willing to do so, they will be redirected to a new QuestionPro survey (which is unlinked to the project survey so that personal data cannot be linked to responses) to register their contact details. Telephone interviews (a minimum of *n* = 10) will be conducted with the co-design and PCG group members who have expressed interest in participation via the Stage 2 survey. Within those that express interest, a level of strategic sampling will be employed to ensure that all participant sector groups are represented within the interview process (e.g., health, education, aged care and community representatives). Research team members will contact the interested participants to provide additional information on the interview process through the provision of a PICF. If the participants decide to participate, they will be asked to return the signed consent form to the La Trobe University researcher so that an interview can be scheduled.

Interviews will be conducted by telephone by a member of the university research team and last approximately 30 min. Participants will be asked to reflect on how the use of the Indigo 4Ms Framework within the co-design process has impacted their understanding of, and/or practices relating to the primary care of older people. Interviews will be audio-recorded with participant permission and transcribed by the research team, which is highlighted in the PICF. Participants will be advised through the PICF and prior to the interview that they can cease participation at any time. They will also be advised that they are able to withdraw their data from the study until 4 weeks post-interview, at which point the data will be de-identified and unable to be distinguished from that of other interviewees. However, participants will be given the opportunity to review their transcripts prior to analysis.

Interview recordings will be transcribed with participant permission and uploaded into a qualitative data analysis program. Using the Braun and Clarke [38] framework, data will be analysed deductively to identify how the use of the Indigo 4Ms framework within the co-design process has impacted the understanding of, and/or practices relating to the care of older people in rural settings. The analysis will also interrogate the potential role of rurality in influencing attitudes and practices relating to the Indigo 4Ms framework among interviewees.

##### Analysis of the Final Model/s of Care

All co-design materials and outputs from the project and findings from the process evaluation will be imported into a qualitative data analysis program (QSR-NVivo) and analysed thematically [38] in relation to where, how, and in what circumstances integration occurs or does not occur in relation to the Indigo 4Ms Framework. As in the previous co-design analysis stages, Braun and Clarke’s [38] framework for qualitative analysis will be used to guide theme identification and development, with evidence of the integration of the initial code used for analysis. The potential role of rurality (e.g., socio-demographic, spatial and resource-related factors) in influencing practices and attitudes to care integration within a 4Ms approach will also be interrogated as a higher-level code.

### 2.8. Data Storage and Handling

All hard-copy data (written notes and consent forms) will be stored in a locked filing cabinet in a locked office at La Trobe University. Electronic data (completed online surveys, transcripts, audio recordings, electronic copies of meeting minutes, Excel files and consent forms) will be stored in a password-protected file on the La Trobe University network, which can only be accessed by research team members, using a password-protected computer. Information provided electronically (which will only be anonymised and aggregated, and not include any identifying information) will be emailed using secure organisational platforms and stored in a password-protected email program. Where consent forms and expression of interest forms are provided electronically to the project manager, these will be forwarded to the La Trobe University research lead using a password-protected email program and saved in the secure La Trobe University password-protected file. At this point, both the sender and the receiver will delete the email from their system and ensure that their email trash is cleared.

No form of identifying data (contact details) will be stored with the returned and completed surveys or interview recordings/transcripts, and raw data will not be accessible to anyone other than the research team members, nor be used for any other project.

Data will be retained for seven years as per the Public Records Office of Victoria protocol in its original form using the storage forms listed above. At the conclusion of this period, hard-copy data will be placed in a secure document disposal bin available at the La Trobe University campus. For electronic data, files will be deleted and disposed of as per the La Trobe University electronic data removal protocols.

## 3. Expected Results

The execution of this intervention and the accompanying process and outcome evaluation will provide important information on the conditions that support the successful co-design of integrated rural primary health models for older people and some of the associated challenges. Specifically, it is hypothesised that the use of the Indigo 4Ms framework within a rural primary care co-design process will be identified as beneficial in fostering perceptions of integration and multidisciplinarity among co-design team members and in developing more integrated models of rural primary care for older people.

## 4. Discussion

This study protocol contributes to the development of a nascent field of study examining how health and aged care services can become more age-friendly [18,19,42,43], with a specific emergent emphasis on the role of co-design in developing 4M approaches to health care for older adults [44]. Specifically, it contributes a rural, international perspective to these debates, which are primarily urban-centric and focused on care for older adults within North America [42,45]. It will achieve this by examining how the use of a 4Ms framework within a co-design process can facilitate or hinder the development of age-friendly integrated care models in a rural context. This will provide information on the conditions that support the successful co-design of age-friendly care models within rural contexts. These findings can then be critically assessed against studies undertaken in urban settings in order to identify rurally specific processes, activities and outputs associated with the co-design of age-friendly care models. This protocol also addresses a significant critique of the current age-friendly health system literature, which relates to the lack of empirical research conducted that examines the design and implementation of the 4Ms framework [19]. Consequently, this protocol will provide evidence to support future research aimed at the co-design of age-friendly, integrated care systems, with specific application to rural contexts. It is anticipated that this protocol can be amended and trialled in other national and international geographic and care contexts.

Importantly, this protocol paper contributes to current policy and practice debates relating to the best-practice design of health and aged care services. In the Australian context, health authorities at both the federal and state levels are promoting the importance of integrated care models in addressing some of the key challenges associated with maintaining the health and wellbeing of older people. A recent research paper prepared for the federal Royal Commission into Aged Care Quality and Safety promotes integrated care as a strategy for overcoming service fragmentation across services and sectors, with the aim of improving health and wellbeing and satisfaction with services among older people. Where care for older people is not integrated, this can lead to the duplication of services, unmet needs, low levels of empowerment and satisfaction with care and increased levels of error within care models [46]. However, research evidence suggests that integrated care works best when it is a bottom-up, community-driven process [47,48], and this protocol provides a set of conditions to be trialled in promoting this type of approach.

Similarly, health and aged care policies across both Australian and international contexts promote the use of co-design methods in ensuring the development of person-centred approaches to care delivery. Evidence from diverse international contexts suggests that the use of participatory design approaches, such as the one being applied in this study, can improve organisational efficiency and improve patient experience [33,49]. This study will contribute to the body of literature examining the efficacy of co-design in rural contexts [50,51] and in relation to the design of services and supports for older people [52,53].

## 5. Conclusions

This protocol outlines the methods for a study identifying how the use of a 4Ms framework within a co-design process can contribute to the development of models of integrated care for older people within an Australian rural region. Specifically, it will identify the processes, activities and outputs that facilitate or hinder the co-design of a 4Ms integrated approach to the care of older people and investigate how the use of the Indigo 4Ms framework within a co-design process facilitates more integrated working practices among multi-disciplinary rural health and aged care workforce and community organisations. These findings will be of significant interest to rural health, aged care and community organisations who are looking to both develop more integrated models of care for older people and increase the use of co-design within service development processes.

## Figures and Tables

**Table 1:** Process evaluation.

**Inputs:** What was contributed (e.g., resources, time, finances or expertise) and from whom; how this changed or evolved across the project	
Project governance	Co-design activity
Characteristics of the project team and PCG group (e.g., sector of work or clinical/community background)	Characteristics of the co-design group (e.g., sector of work or clinical/community background)
Scheduling and level of attendance at PCG meetings	Scheduling and level of attendance at each co-design workshop
Financial and other resources allocated to the project	Financial and other resources allocated to the project
**Activities:** What activities were completed, and when, where, why and how, how this aligned with project plans and how the Indigo 4Ms were discussed within project activities	
Project governance	Co-design activity
Numbers and frequency of PCG meetings	Numbers and frequency of co-design groups
Topics within/content discussed within PCG meeting agendas and minutes	Topics within/content of written notes generated by the co-design groups and research team
**Outputs:** What was generated and how this aligned with project plans; how outputs were representative of the Indigo 4Ms approach	
Project governance	Co-design activity
Ratified project management documents (project plans, governance plans, terms of reference and governance plans)	Reports generated using data from the co-design group activities that have been ratified by the PCG
Written outputs generated by the project team (reports to funding agency and scoping papers)	

## Data Availability

Not applicable.
